# The value of publishing case reports for the professional development of Chinese residents: a qualitative study

**DOI:** 10.1186/s12909-025-07459-2

**Published:** 2025-07-01

**Authors:** Zhihui Yang, Wen Chen, Ruoyu Li, Xiaowen Wang, Mi Yao

**Affiliations:** 1 Department of Dermatology and Venerology, Peking University First Hospital, No.8 Xishiku Street, 100034 Beijing, China; 2 National Clinical Research Center for Skin and Immune Diseases, Beijing, China; 3 Beijing Key Laboratory of Molecular Diagnosis of Dermatoses, Peking University First Hospital, Beijing, China; 4 NMPA Key Laboratory for Quality Control and Evaluation of Cosmetics, Peking University First Hospital, Beijing, China; 5 Department of General Practice, Peking University First Hospital, No.8 Xishiku Street, Xicheng District, Beijing, 100034 China

**Keywords:** Case report, Residents, Graduate medical education, Qualitative study

## Abstract

**Background:**

In comparison with high-income countries, medical education resources in low- and middle-income countries (LMICs), such as China, are severely lacking. In China, residents' clinical work is characterized by extensive demands and mechanized approaches, resulting in a paucity of opportunities for active thinking and learning during clinical rotations. Moreover, these residents lack the time and experience necessary for scientific research training. However, these countries possess a wealth of case resources, including numerous classical and complex cases that hold significant teaching and publication value. The effective utilization of this advantage and the integration of case reporting as a distinctive graduate medical education (GME) platform remain to be explored.

**Methods:**

The study was conducted between September 2023 and March 2024 in Beijing, China. Purposive sampling was used to recruit 15 residents who were in the first or second stage of standardized residency training and had successful experience in publishing case reports. Semi-structured in-depth interviews were conducted on a one-on-one basis to explore the role of case reports in the professional development of residents in LMICs. Thematic analysis was employed to analyze the data.

**Results:**

Of the 15 participants, 3 were male and 12 were female, with an average age of 27.2±2.6 years. 12 subthemes were extracted, from which 5 themes were identified, including clinical professional competency, scientific research competency, collaborative and communicative competency, sources of pressure and implementation evaluation. In terms of clinical competency, publishing case reports can increase residents' basic medical knowledge reserve, practical ability of diagnosis and treatment, and self-learning in daily clinical practice. In terms of scientific research competency, the case report can guide young residents to commence their academic careers, improve academic writing skills and strengthen their initiative in scientific research. Furthermore, it has been demonstrated that case reports can enhance awareness and skills in multidisciplinary collaborations, as well as communication with patients, supervisors and journal editors/reviewers. Heavy clinical responsibilities during residency training are common external pressures while anxiety is a common internal pressure during case report writing. Feasible aspects of integrating case report training into GME in LMICs include the relatively low time commitment and abundant case resources. However, challenges include inconsistent training and assessment standards.

**Conclusions:**

It is suggested that case report training be specially integrated into GME in LMICs with sufficient valuable case resources and capacity to manage complex cases, based on the value of case report experience in the career development of residents and the current situation of medical education in these countries.

**Supplementary Information:**

The online version contains supplementary material available at 10.1186/s12909-025-07459-2.

## Background

A case report constitutes a detailed account of the symptoms, signs, diagnosis, treatment, and subsequent follow-up of one or multiple patients, typically encompassing unusual case circumstances or innovative treatment regimens [1]. The publication of case reports can facilitate the acquisition of invaluable expertise in the diagnosis and treatment of complex diseases, generate novel diagnostic and therapeutic concepts, and even stimulate the advancement of academic domains by unveiling novel research directions [1, 2]. However, due to the rarity of most cases, findings are often difficult to replicate, and the cause-effect relationship is hard to establish, which may lead to over-interpretation [3–5]. Consequently, the evidence-based level of case reports is low, and their popularity among researchers is gradually declining [6].

While the scientific value of case reports remains a contentious issue [4, 7], there is growing recognition of their educational value. From a clinical standpoint, the completion of a case report has been shown to effectively promote clinical practice participation, train clinical skills such as collecting a medical history, summarizing case characteristics, and case presentation [8–10], and promote diagnostic excellence by improving the quality of the diagnostic reasoning process [2]. Furthermore, the case management allows residents to understand the complexity and variability of clinical situations [11], which cultivates doctors’ confidence in identifying rare diseases and dealing with treatment complications [5]. From a scientific research perspective, case reports, as the first publications for most young residents at the start of their careers [4, 5, 12, 13], have been shown to greatly enhance their skills in literature searching, reviewing, and academic writing, increase their experience with journal submission and peer review [6, 9, 13–15], and thereby pave the way for their transition into the realm of medical academic writing [11, 14]. Case reports also serve as valuable presentation materials for most low-seniority residents, facilitating their participation in academic conferences and honing their presentation skills [16]. Furthermore, it fosters critical thinking skills [8, 9], which are paramount for future research endeavors. Besides, the process of completing the case report emphasizes networking and collaboration [8, 9], including communication with the patient to obtain critical information on medical history and informed consent [2], and liaising with the supervisor and reviewer [11, 13]. From a utilitarian perspective, the publication of the article also enhances the academic curriculum vitae (CV) [8, 9]. A qualitative study conducted in Japan specifically has demonstrated the value of the case report-driven medical education in rural family medicine education [11]. However, the practical effect and extensibility of this educational method in China and other LMICs remains to be ascertained.

Currently, the concept of medical training in China and the majority of LMICs is aligned with that in Western developed countries, such as the common program requirements of the Accreditation Council for Graduate Medical Education in the United States and the Canadian Medical Association framework. These teaching concepts place significant emphasis on competency-based education and scholarly activities [8, 17]. However, in an LMIC such as China, there is a severe paucity of clinicians and a shortage of medical education resources, including supervisors and training sites. Consequently, the clinical work of residents is tedious and mechanized. During clinical rotations, residents frequently demonstrate a lack of engagement in problem-solving and active learning, a paucity of experience in scientific research training, and a significant burden of physical and mental pressure [18]. It is evident that there is a considerable discrepancy between the expectation of medical education and its practical outcomes [19]. The imbalanced distribution of medical education resources and the bewildering medical education pathways further exacerbate the disparities in residents’ medical expertise [20]. This underscores the need to enhance the residency training methodologies employed in LMICs, with a view to promoting competencies that are both clinical and scientific in nature.

In contrast to Western countries, China and other LMICs possess a distinct advantage in their extensive case resources, which include valuable and challenging cases. These resources provide a strong foundation for case-report training as a medical education platform in LMICs. Additionally, the concise nature of case reports allows for efficient completion, aligning with the modern educational focus on maintaining a healthy work-life balance and ensuring adequate rest for residents [21, 22].

## Objectives

The present study adopted a qualitative research method, conducting in-depth interviews with residents who had experience of publishing case reports. The specific experiences and perspectives on publishing case reports were explored to understand the role and value of case reports in the professional development of residents. The study aimed to explore whether case reports could become a distinctive training platform for residents in LMICs, and to provide suggestions for improving the graduate medical education (GME) program.

## Methods

### Study design and aim

Writing case reports involves clinical practice, scientific research, communication, and collaboration. To better understand the value of case reports in residents’ professional development, we developed a theoretical framework grounded in adult learning principles and experiential learning theories.

Firstly, experiential learning theory posits that learning occurs through a cyclical process of experience, reflection, conceptualization, and experimentation. In our study, residents engaged in the exploration of real cases, which facilitated repeated iterations between practice, learning, and exploration. This process enabled the transformation of experience into knowledge, aligning with the principles of experiential learning [23].

Secondly, adult learning principles emphasize the importance of self-directed learning, relevant to the learner’s experience and motivation [24, 25]. Our training platform was designed to provide residents with opportunities to realize their potential, build their confidence, and increase their motivation for active engagement. By applying adult learning theories, we were able to understand the residents’ learning motivation and behavior more effectively.

Through this theoretical framework, we employed a systematic and iterative approach in our qualitative research to identify the value of case reports in residents’ professional development. This framework not only guided the study design and data analysis but also provided a robust foundation for interpreting our findings.

An in-depth qualitative exploratory study was conducted utilizing semi-structured one-on-one interviews with residents from 5 tertiary hospitals in China, including Peking University First Hospital, China-Japan Friendship Hospital, Xuan Wu Hospital, Beijing Hospital, and The First Affiliated Hospital of Zhengzhou University. The study aimed to investigate the value of publishing case reports in the professional development of residents in LMICs.

### Setting and participant recruitment

The study included residents who: (a) were in the first or second stage of standardized residency training; (b) had successful experience of case report publication. Residents who had completed residency training prior to the publication of case reports were excluded. A purposive sampling method was employed to include a diverse cohort of participants varying in age, gender, medical specialty, and experience level in case report writing. 26 residents were invited to participate via email or mobile application, WeChat, of whom 9 declined without response and 2 were excluded according to the exclusion criteria. The recruitment and interview process was conducted iteratively in conjunction with the data analysis. Interview scheduling was terminated when the data analysis revealed significant replication of themes, with no new information or themes identified, indicating that data saturation had been reached.

### Instruments

The Tencent Meeting was used to conduct online interviews when participants could not attend in person. Suishenglu software, a recording device, was used to capture the entire conversation and transcribe the recordings into text. The qualitative research software NVivio 14.23.2 was used to facilitate data management and analysis.

### Data collection

Semi-structured interviews were conducted by the first author (ZY) in Beijing, China, between September 2023 and December 2023. Six participants had previous experience of working with the interviewer (ZY), an assistant research fellow at Peking University First Hospital, and were already acquainted with each other. However, none of the participants had a teacher-student or administrative subordinate relationship with the researchers, nor had they collaborated with the researchers in writing case reports or other academic articles. The interview topic guide was developed and modified following consultation with experts in the fields of academic writing and medical education. Two pilot interviews were conducted to ensure that the questions focused on the experience and perception of case report publication. The finalized interview outline is presented in Appendix A.

Prior to the formal interview, the interview outline was sent to the interviewees via instant messaging software, and the scheduled interview time was communicated. At the commencement of the interview, the interviewer expounded on the objective and methodology of the research to the interviewees. The interviewer further clarified the necessity of recording the interview content and obtained the interviewees’ explicit consent. Demographic information was collected from participants prior to each interview. Interviews were conducted in a relaxed environment, and brief icebreaker activities were used to reduce participants’ anxiety. In accordance with the guidelines set forth in the interview outline and the actual circumstances of the interviews, the interviewer made flexible adjustments to the sequence of questioning, and pursued pertinent inquiries appropriately. The entire interview process was recorded and transcribed verbatim into text data within 24 h of the interview taking place. Data saturation was attained after the completion of 13 interviews, after which two further residents were interviewed to ensure comprehensive data coverage. The average duration of each interview session was 34.5 ± 12.5 min.

### Data analysis

The data analysis was conducted between September 2023 and March 2024. The study adopted an inductive analytical approach to analysis, enabling themes to emerge directly from the data. On completion of each interview, two researchers (ZY and WC) carried out transcription and examined their transcripts for errors by listening back to the audio-recording and reading the transcripts simultaneously.

Three researchers—two (ZY and WC) with MDs in Dermatology and one (MY) with a PhD in General Practice—all with extensive experience in qualitative research, independently read the transcripts several times to understand the text and then coded the transcripts line-by-line. This diversity in background and expertise helped to bring multiple perspectives to the analysis, reducing the likelihood of a single dominant viewpoint influencing the findings.

The coding process followed the six-step framework for thematic analysis outlined by Braun & Clarke [26], including familiarization with the data, generating initial codes, generating themes, reviewing potential themes, defining and naming themes, and producing the report. To ensure intercoder reliability (ICR), as recommended by O’Connor & Joffe [27], the researchers engaged in regular meetings to discuss coding decisions, resolve discrepancies, and reach consensus. This iterative and collaborative process enhanced the credibility and trustworthiness of the thematic analysis. Acknowledging the inherent influence of researcher perspectives and biases, we employed reflexivity throughout the study, regularly reflecting on how our assumptions might shape interpretations.

Discrepancies in data analysis were resolved through a structured process. When disagreements arose, the three researchers (ZY, WC, and MY) engaged in a detailed discussion to revisit the transcript together and understand the rationale behind each coding decision. This step was crucial for identifying the root causes of the discrepancies. If consensus could not be reached through initial discussions, a fourth investigator (XW) was consulted. This external perspective helped to provide additional insights and facilitate a resolution. The researchers then revisited the relevant sections of the transcripts and reviewed the coding decisions in light of the new insights gained from the consultation. This iterative process ensured that all perspectives were considered and that the final coding decisions were well supported by the data. Throughout the process, all discussions and decisions were meticulously documented to ensure transparency and traceability. This documentation included the nature of the disagreements, Cohen’s kappa for measuring ICR, the rationale for the final coding decisions and any changes made to the coding scheme as a result of the consensus-building process.

The results of the data analysis, including themes, subthemes, interpretations and quotations, were provided to two interviewees for review and feedback. No significant objections were raised by the interviewees, thereby confirming the credibility and authenticity of the study’s conclusions.

### Rigor and trustworthiness

The criteria for the trustworthiness included credibility, transferability, dependability, and confirmability [28, 29]. Specifically, credibility was enhanced through member checking, where findings are shared with study participants for feedback and validation, ensuring that the interpretations accurately reflect their perspectives. Peer debriefing was also conducted to ensure analytical rigor. Transferability was facilitated by providing rich, detailed descriptions of the context and participants, allowing the applicability of findings to be assessed in other settings. Dependability was maintained through detailed documentation of the data collection and analysis process, including an audit trail of decisions made throughout the thematic analysis. Finally, confirmability was supported by employing a team-based approach to data analysis, allowing for multiple interpretations and reducing researcher bias. These strategies collectively aimed to ensure the trustworthiness and rigor of the findings.

## Results

A total of 15 residents participated in this study, including 3 (20%) males and 12 (80%) females, with an average age of 27.2 ± 2.6 years. 8 were in the first stage of standardized residency training and 7 were in the second stage of standardized residency training. The clinical specialties of the respondents included dermatology and venerology (7), surgery (2), internal medicine (2), oncology (1), interventional ultrasound (1), and Chinese and western integrative medicine (2). Demographic characteristics, experience of publishing case report, and interview duration for the 15 study participants are presented in Appendix B. 241 initial codes were generated from a total of 517.5-minute interview. 12 subthemes were extracted from these initial codes, and through further analysis, 5 themes were identified, including clinical professional competency, scientific research competency, collaborative and communicative competency, sources of pressure, and implementation evaluation (Table 1).

**Table 1:** The study themes and Sub-themes

Themes	Sub-themes
Clinical professional competency	The basic medical knowledge reserve
	Practical ability of diagnosis and treatment
	Self-learning habit in daily clinical practice
Scientific research competency	Commencement of the academic career
	Academic writing skills
	Initiative in scientific research
Collaborative and communicative competency	Multidisciplinary collaborations
	Communication ability
Sources of pressure	External pressure
	Internal pressure
Implementation evaluation	Implementation feasibility
	Implementation challenges

### Theme 1: clinical professional competency

#### Sub-theme 1.1: the basic medical knowledge reserve

The process of writing case reports significantly contributed to the accumulation of basic medical knowledge. This occurred at all stages of case report preparation, from inception to publication.

a. Knowledge obtained in determining the publication value of a case.

Identifying clinical cases for reporting involved reviewing previous literature. This process not only helped assess the case’s publication potential but also fostered an understanding of related diseases. *“My mentor encountered several interesting cases and suggested I write them up for publication. However*,* upon reviewing the literature*,* I discovered that many similar articles had already been published*,* diminishing the publication value of these cases. Since that experience*,* whenever my mentor identified an intriguing case*,* I would first check the literature to ensure its publishability before writing it up.” (#5*,* female*,* aged 33 years)*.

b. Learning during doctor-patient communication.

Before communicating with patients, residents needed to undertake a lot of learning to consolidate their professional knowledge, aiming to express ideas clearly to patients and gain their trust for effective collaboration in diagnosis and treatment. *“When communicating with the patient*,* I often struggled with professional questions from patients*,* which made patients gradually lose confidence in my professional abilities and didn’t want to continue with the treatment.” (#10*,* female*,* aged 27 years)*.

c. Learning in the diagnoses and treatment process.

Clear diagnoses and optimal treatment strategies are crucial for case reports, which require essential examinations. However, this was reported to be difficult for many young residents who had little knowledge of the rare cases. Consequently, they must systematically acquire the knowledge necessary to perform the diagnostic, differential, and therapeutic aspects of the disease, thereby ensuring that all crucial laboratory or imaging examinations are prescribed. *“When I submitted the manuscript*,* a major issue raised by reviewers was that I forgot to stain a biomarker in the tissue. Actually*,* at the time*,* I lacked the requisite knowledge regarding the significance of this biomarker in the disease diagnosis. However*,* since I was unaware of the necessity to stain it at the time*,* I can’t rectify it now.” (#8*,* female*,* aged 29 years)*.

#### Sub-theme 1.2: Practical ability of diagnosis and treatment

a. Accumulating clinical practice experience.

In addition to the expansion of basic medical knowledge reserve, problem-oriented learning further strengthened the memory of the clinical experience of the case, thereby enhancing the ability to diagnose and treat the case-related disease in the future. *“The main motivation for writing case reports was to organize my knowledge about the related disease. Once I completed a case report*,* I gained a deeper understanding of the disease. The next time I encountered patients with similar clinical manifestations*,* I would be able to diagnose them instantly.” (#5*,* female*,* aged 33 years)*.

b. Training medical thinking.

Although case reports focus on rare cases and the knowledge gained may not directly apply to daily practice, residents believed that writing them trained diagnosis and treatment thinking, enhancing logical reasoning. *“Indeed*,* writing a case report offered minimal assistance in improving my clinical diagnostic capabilities because the scenarios in the reports are rarely encountered in practice.” (#7*,* female*,* aged 28 years) “The completion of a case report facilitated the acquisition of skills necessary for managing complex cases. When faced with similar cases in the future*,* I would refer to the treatment plan developed in the case report*,* prescribing examinations and treatments step by step.” (#3*,* female*,* aged 27 years)*.

c. Developing a responsible treatment attitude.

The success of publishing case reports also served to stimulate the interest of residents in publishing more case reports. To this end, residents deliberately collected case data of particular significance in practice. When faced with complex cases, residents actively conducted examinations instead of merely referring patients to senior clinicians. They followed up on prognoses and adjusted treatment plans based on examination results and drug efficacy. This fostered meticulous attention to patients’ conditions and underlying causes, promoting a responsible attitude toward diagnosis and treatment. *“Having the experience of writing a case report*,* I would always keep a question in my mind during clinical rotation*,* namely ‘Is the case I encountered suitable for publication?’ For cases with an unclear diagnosis*,* I actively consulted literature*,* considered possible diagnoses*,* ordered further tests*,* and monitored results closely. This not only helped clarify diagnoses for patients but also helped me develop a responsible habit in practice*,* rather than referring difficult cases elsewhere.” (#11*,* female*,* aged 26 years)*.

#### Sub-theme 1.3: Self-learning habit in daily clinical practice

Successfully publishing a case report signified more than accumulating disease knowledge; it significantly impacted a physician’s long-term learning capacity. This experience equipped residents to manage complex cases independently, fostering confidence and motivation to further explore clinical practice while cultivating independent thinking and problem-solving skills. *“Writing case reports is an effective way to develop good habits. When challenges arose*,* we took the initiative to find answers rather than just following orders. This involved conducting literature searches and consulting experts in other disciplines*,* enriching our clinical knowledge and refining our thinking and exploration skills.” (#15*,* male*,* aged 28 years)*.

### Theme 2: scientific research competency

#### Sub-theme 2.1: commencement of the academic career

a. An understanding of the scientific research process.

Junior residents often published case reports as their first articles. They believed case reports shared similarities with original research and reviews regarding the scientific research process—including literature search, academic writing, submission, and revision. This experience equipped them for future publications in peer-reviewed journals, laying the groundwork for more complex research. *“Although it was just a case report*,* I gained a lot from the process*,* from writing to submitting and publishing. The steps for writing original articles would be similar*,* providing a foundation for future scientific research.” (#13*,* female*,* aged 25 years)*.

b. Crossover with original research.

Although the methodologies employed in experiment and clinical research were considerably more intricate than those in case reports, the diagnosis of many case reports was contingent upon laboratory examinations utilizing the latest technology, thereby enabling residents to acquire learning opportunities of experimental operations. *“My patient’s unique infection required detecting potential immune gene defects to investigate the etiology. Thus*,* I had the opportunity to assist my senior colleague in collecting the patient’s DNA for sequencing*,* which provided me with hands-on experience in fundamental experiments.” (#2*,* female*,* aged 24 years)*.

Concurrently, given that the subjects of case reports and clinical studies were individuals and groups, respectively, some residents believed the case reports were, in essence, the epitome of case series studies or other descriptive clinical studies. *“My supervisor encountered an unusual case during surgery and asked me to document it for publication. However*,* our hospital’s records showed more than ten similar cases*,* allowing me to conduct a case-series study.” (#11*,* female*,* aged 26 years)*.

c. Confidence in scientific research.

Due to the abundance of clinical case materials and the short length of case reports, the resources and efforts required to write case reports are significantly lower compared to other research types. Thus, this type of research could help novice residents develop confidence in publishing articles at a minimal cost. *“After publishing one case report*,* I found publishing articles was not as challenging as I imagined. This experience boosted my confidence and motivated me to pursue further research*,* knowing my outcomes would be published as long as I committed to overcoming challenges.” (#11*,* female*,* aged 26 years)*.

#### Sub-theme 2.2: academic writing skills

a. Generalization and summarization.

Many junior residents found writing their first case report time-consuming and demanding, requiring significant effort to reduce their manuscript’s word count to meet journal criteria. As a result, this experience fostered the skills in capturing clinical and scientific research priorities. *“The biggest challenge I faced in writing the case report was including too many unnecessary details*,* which made it resemble a complete medical record. However*,* keeping the report concise was crucial*,* especially since many international journals had reduced their word limits to 300–600 words. Every sentence had to be meaningful and support the central idea. Summarizing case characteristics and current research progress was very challenging*,* but I gradually mastered it after a couple of training sessions.” (#2*,* female*,* aged 24 years)*.

b. Logical thinking.

Many residents believed the logical thinking required for writing case reports was essentially similar to the one required for original research. Despite the substantial discrepancy in word count, a case report also significantly enhanced their writing proficiency. *“Publication success depends heavily on innovation and efforts*,* whether it is an original article or a case report. Clearly and logically explaining the case’s innovation*,* its similarities and differences with existing literature*,* and possible reasons in limited words were lessons I learned from writing case reports.” (#10*,* female*,* aged 27 years)*.

c. Language expression.

Despite the brevity, case reports still serve as scientific research articles, exercising the application of academic language. Notably, for medical residents in non-English-speaking countries, each experience in English article writing enhanced their academic English expression abilities. *“At first*,* my writing style wasn’t as polished as that of native speakers. A senior fellow helped refine my manuscript*,* making it resemble a scientific research paper. Initially*,* I learned through imitation*,* and my English writing style gradually mirrored his.” (#15*,* male*,* aged 28 years)*.

#### Sub-theme 2.3: initiative in scientific research

a. Scientific research awareness.

The publication experience of publishing case reports enriched the academic CV of residents, thereby enhancing their awareness and enthusiasm for academic publication. Concurrently, as academic novices, they realized their lack of research skills and the importance of increasing scientific research experience during the process of case reporting, which further increased their scientific research awareness. *“I will undoubtedly pursue more research in the future. Each article I wrote trained my writing skills. Besides*,* papers are always important at any career stage. Whether original research or case reports*,* if published in the top journal*,* can significantly enrich my resume.” (#9*,* female*,* aged 26 years)*.

b. Habit of accumulating scientific research resources.

While junior residents typically relied on supervisors to identify valuable cases, as they gained more experience publishing case reports, some had developed the habit of independently collecting clinical cases. This proactive approach helped discover publishable cases, contributing to the scientific research resource pool and encouraging independent scientific exploration.*“My mentor always encouraged me to focus on collecting clinical data when encountering valuable cases. This practice was not only for writing case reports but also for future cohort or case-series studies. Thus*,* I meticulously documented significant cases during my clinics and did identify several suitable cases for publication in the process.” (#7*,* female*,* aged 28 years)*.

Conversely, the lack of scientific research consciousness has consequently resulted in the “difficult birth” of scientific research. In the course of recruiting respondents, we found that surgeons exhibited a marked paucity of experience in publishing case reports when compared with doctors in other specialties. Moreover, surgeons themselves acknowledged that this shortage was associated with their inadequate scientific habits. *“Surgeons in our hospital rarely published case reports. This might be due to a lack of cases with sufficient publication value. It was also possible that we lacked the habits of thoroughly investigating complex cases*,* such as tracing the results of genetic testing to assist patients in identifying the underlying etiology. As a result*,* it was difficult to encounter complete and valuable case data.”(#11*,* female*,* aged 26 years)*.

c. Ability to lead the project progression.

As a newly entered resident, a case report is generally the first study where the resident takes a lead role to prompt the whole process, with mentors acting as assistants. Since the tutor lacked sufficient time to personally supervise every simple case report and case reports were generally manageable for beginners, residents were allowed to demonstrate autonomy in scientific research while boosting their confidence in the process.*“In my personal experience*,* the role of residents in writing the case report was much more significant than that in other types of research. In large-scale multicenter studies*,* I played a minimal role*,* merely executing the leaders’ ideas. Nevertheless*,* in the process of completing case reports—from collecting medical history to writing the article—my involvement was much greater. While supervisors were generally willing to help*,* they were preoccupied with larger studies*,* limiting their availability for case reports.” (#15*,* male*,* aged 28 years)*.

### Theme 3: collaborative and communicative competency

#### Sub-theme 3.1: multidisciplinary collaborations

a. Enhancing the awareness of multidisciplinary cooperation.

From a clinical perspective, many residents expressed a preference for collaborative approaches when addressing complex cases, as this strategy enhanced diagnostic accuracy and identified optimal treatment strategies. Such experiences fostered a heightened awareness of multidisciplinary diagnosis and treatment.

*“In the case report I wrote*,* we misdiagnosed the patient initially as having bronchiolitis obliterans based on CT images. However*,* after consulting with experts from imaging and pathology*,* we revised the diagnosis to diffuse panbronchiolitis. This experience highlighted the importance of multidisciplinary team (MDT) collaboration. Our department had established a standard protocol for analyzing image data*,* which was followed by experts and residents alike. This practice may*,* in some cases*,* limit the breadth of our thinking*,* leading to misdiagnosis. Thus*,* we need to seek opinions from the imaging department*,* who may provide different views and enhance our diagnostic accuracy.” (#15*,* male*,* aged 28 years)*.

From a scientific research perspective, many formerly recognized specialized diseases have been revealed as local manifestations of systemic diseases. To elucidate disease correlations and promote advancements in medical research, multidisciplinary thinking is crucial; and to broaden academic horizons, multidisciplinary cooperation is essential. *“In the future*,* I would like to explore collaborative research opportunities across disciplines. For example*,* asthma*,* allergic rhinitis*,* and atopic dermatitis*,* which belong to respiratory medicine*,* otorhinolaryngology*,* and dermatology*,* respectively*,* could be studied together. By collaborating*,* we could gain a more comprehensive understanding of these conditions and advance our research more meaningfully.” (#15*,* male*,* aged 28 years)*.

b. Establishing collaborative connections.

The complexity of cases often required MDT involvement, providing residents with opportunities to connect with experts from diverse disciplines. This experience established a foundation for future multidisciplinary collaborations in both clinics and research. *“My interactions with clinicians from various departments enriched my knowledge in their fields. I am deeply grateful for the connections I’ve established through this experience.” (#12*,* female*,* aged 28 years)*.

#### Sub-theme 3.2: communication ability

a. Communicating with patients.

The publication of a case report necessitates a more comprehensive medical history than is typically recorded in the daily clinic. Residents must also obtain informed consent from patients before publication, highlighting the importance of effective communication. However, the potential for contradiction between doctors and patients in LMICs serves to compound the complexity of communication. In such contexts, residents came to recognize that a responsible approach toward patients was indispensable for successful communication. *“I reported a case of a skin foreign body granuloma. I communicated with the patient 4–5 times to determine the exact foreign body causing the skin lesions. The patient was very busy with his work and sometimes impatient. At this point*,* I explained that my follow-up was out of concern for his health*,* not just for publishing the case report.” (#1*,* female*,* aged 27 years)*.

b. Communicating with supervisors.

Residents often relied on their supervisors for guidance when facing challenges, but supervisors could be too busy to address queries. In such contexts, residents learned how to efficiently and clearly express their problems and obtain effective advice. *“There was a misunderstanding during my case report submission. I was asked to submit my manuscript to the main journal of one medical journal*,* but I mistakenly submitted it to the case-report sub-journal of that journal. I believe this was due to insufficient communication. In retrospect*,* I found that when I had a clear appointment with my supervisor and we focused on communication*,* problems could be solved more efficiently. Conversely*,* when I left messages with my supervisor*,* even if the questions were clearly listed*,* he sometimes misunderstood my questions and neglected some of my queries.” (#1*,* female*,* aged 27 years)*.

c. Communicating with journal editors and reviewers.

When communicating with editors/reviewers over the revision of case reports, residents should not only recognize the opinions of reviewers, but also adhere to their own arguments, reflecting their own professionalism. The residents gradually learned how to grasp the “degree” in communication and how to express the above views clearly and logically at the same time during the revision. This process significantly trained the resident’s professional written communication ability. *“I was generally humble and courteous in my written communication with the editors/reviewers*,* acknowledging their comments*,* expressing gratitude*,* and providing detailed explanations in response. Occasionally*,* I felt that some questions raised by the editor/reviewer were not professional enough and contradicted my opinion. In those instances*,* I respectfully acknowledged their opinions while maintaining my perspective and articulating the reasons behind our differing views.” (#3*,* female*,* aged 27 years)*.

### Theme 4: sources of pressure

#### Sub-theme 4.1: external pressure

In LMICs like China, resident physicians frequently face pressure from demanding clinical responsibilities that significantly cut into their research time, delaying the writing of case reports. *“When I was a master’s student*,* I had fewer clinical tasks and was proactive in writing case reports. However*,* in my current doctoral phase*,* my focus has shifted to clinical work and my dissertation. I often find myself finishing clinical duties until midnight*,* leaving me exhausted and with lumbar swelling and nerve compression*,* which forces me to postpone my case report writing tasks.” (#7*,* female*,* aged 28 years)*.

#### Sub-theme 4.2: internal pressure

Case reports often serve as the initial articles for resident physicians at their commencement of academic careers, introducing a duality of emotions:

a. Anxiety due to lack of experience.

Residents often experience confusion and anxiety due to their lack of research experience at their first time time writing case reports. However, since the complexity level of the case report is generally within the capabilities of novices, when residents become more familiar with the process of writing case report, the anxiety diminishes significantly. *“I had never published an article before*,* so each step of writing the case report—from data collection to submission—was a process of exploration for me*,* filled with anxiety and confusion. However*,* after my first publication*,* I realized writing case reports is quite simple.”(#2*,* female*,* aged 24 years)*.

b. Anxiety due to the urge of self-fulfilling.

Residents might experience pressure and nervousness stemming from a desire to demonstrate their capabilities in an unfamiliar professional environment. However, the pressure could also serve as a motivation for improved performance and skill acquisition. *“This was the first task my supervisor assigned me after I joined the department. I place great importance on my supervisor’s first impression of me. So*,* I really wanted to excel in this endeavor*,* which made me particularly anxious and stressed.”(#11*,* female*,* aged 26 years)*.

### Theme 5: implementation evaluation

#### Sub-theme 5.1: Implementation feasibility

a. Low time commitment.

Many residents reported that heavy clinical assignments and graduation dissertation requirements were primary sources of stress, necessitating considerable time investment and leaving them sleep-deprived. Compared to clinical or basic science research as required for graduation, case reports were shorter and could often be completed during fragmented time in clinical work. *“Given the concise nature of the case report and its close alignment with clinical work*,* the writing process of case report only takes approximately 1–2 weeks from the initial conception to manuscript submission.” (#6*,* male*,* aged 30 years)*.

b. Abundance in the case resources.

Many residents felt anxious about publishing their graduation theses due to factors such as limited project funding and outcomes that did not meet expectations. Conversely, case reports did not face these issues. With an abundance of clinical cases available, publishing case reports was much easier, provided the case was distinctive enough. “*I was under great pressure to do clinical research before. On one hand*,* the data quality was subpar*,* requiring repeated quality control and data supplementation. On the other hand*,* the research results were illogical*,* necessitating constant changes to the statistical methods. However*,* these concerns are irrelevant when writing case reports*,* especially in LMICs*,* where there is a high volume of valuable cases.*” *(#2*,* female*,* aged 24 years)*.

#### Sub-theme 5.2: implementation challenges

a. Inconsistent training standards.

The format and word count requirements for case reports across journals are inconsistent. Some journals require brief image descriptions of around 300 words, while others accept longer reports with an introduction and discussion. The focus of different case reports also varies, with some emphasizing unique diagnoses and others highlighting innovative treatments. These differences are mainly influenced by the characteristics of the cases encountered and have a high degree of randomness, leading to considerable variability in the workload for completing the case reports, complicating standardization in training. *“Cases with unique pathological findings may not require direct patient contact*,* as supervising doctors might have gathered the necessary information. However*,* innovative treatment cases often require long-term follow-up*,* which tests the resident physician’s communication skills and clinical competence during patient interactions.”(#7*,* female*,* aged 28 years)*.

b. Challenges in assessing training effectiveness.

The level of published case reports is influenced by various confounding factors, including the specificity of the case and luck, making it unfair to judge training outcomes solely based on the journal’s impact factor in which the case is published. Thus, assessing the effectiveness of the training objectively and uniformly is challenging. A more appropriate evaluation of training effectiveness might focus on the clinical knowledge gained related to the case and the development of research writing skills.*“Although I have experience publishing an image report in the British Medical Journal*,* I also possess several cases that I believe have comparable reporting value but have been rejected by multiple journals and remain unpublished to date. Thus*,* assessing training outcomes based on journal impact factors may be inappropriate*,* as publication in high-impact journals often depends on luck—such as encountering a valuable case and having a reviewer interested in the topic. Instead*,* the benefits gained from writing case reports*,* such as improved understanding of related diseases and enhanced writing skills*,* should be prioritized in the evaluation process.”(#5*,* female*,* aged 33 years)*.

## Discussion

This study utilized in-depth interviews with Chinese residents who had published case reports to explore the impact of completing case reports on the professional development of residents. Aligned with previous research [2, 6, 8–11, 13–15], the study’s findings indicated that the experience of publishing case reports played a significant role in the cultivation of clinical professional, scientific research, collaborative and communicative competency of residents. The sources of pressure during the case report completion were explored and the feasibility and challenges of implementation were assessed. The findings suggested that case reports played a valuable role in the training of residents and offered significant insights for the promotion of case report training within the GME platform in LMICs with sufficient case resources.

In previous studies, the value of case reports in medical education was primarily centered on the accumulation of scientific research experience and the enhancement of scientific research skills, including enriching academic CV [12, 13], gaining hands-on experience in producing medical publications [13], and improving academic writing skills [30]. Besides, previous studies have demonstrated the value of case reports in the patient management process, particularly with regard to the experience of how to deal with difficult cases [3, 11]. The values were also validated in this paper. Furthermore, this study underscored the merits of case reporting in comparison to conventional teaching methodologies for enhancing clinical proficiency, which was evidenced by a solid foundation of fundamental knowledge and the adeptness in clinical diagnosis and treatment. This is due to the fact that the nature of case reporting is aligned with case-based learning (CBL) [6, 11], a pedagogical approach that fosters active learning [31]. During the completion of the case report, residents have the opportunity to independently interact with authentic cases, analyze various differential diagnoses or pathogenesis hidden behind the unusual clinical manifestations, apply the learned theories from literature to the diagnosis and treatment of the cases, perform investigations to confirm the diagnosis/etiology of cases, and formulate the next diagnosis and treatment plan. This series of learning processes, ranging from memory, understanding, analysis, evaluation, and creation, emphasize higher-order thinking skills [32, 33]. Furthermore, case reporting demands a higher level of integration between clinical practice and literature theory learning when compared with traditional CBL. This integration further strengthens the training of critical thinking. Concurrently, case reporting is also a form of experiential learning, the efficacy of which is directly proportional to the degree of engagement [23]. In comparison to conventional clinical rotations, case reports offer a more substantial involvement for residents [11], leading to a notable enhancement in their experiential learning. In the present study, many residents responded that they had a longer memory of the disease knowledge they learned during case reporting and were more skilled in applying it to future diagnoses and treatments.

Moreover, this study indicates that the experience of case reports serves not only to augment residents’ clinical and scientific research experience, but also to cultivate their awareness and initiative to engage in independent learning, scientific research, and team cooperation from a long-term perspective. Based on the adult learning principles in humanistic psychology [33, 34], learning is an act of self-actualization and ongoing personal development, the humanistic approach to learning is student-centered, promoting students’ own needs, meeting their expectations, and emphasizing their responsibility, initiative and self-orientation [33]. In the present study, the experience of publishing case reports stimulated the residents’ research interest, the need to accumulate clinical experience and the expectation to further enrich their CV [8, 9]. This, in turn, significantly enhanced their motivation to publish more case reports and original studies. Concurrently, the residents’ growing confidence in managing complex cases and in scientific research writing further facilitated the translation of this motivation into tangible actions [5]. For instance, in order to ascertain the publication value of challenging cases encountered in daily clinics, residents actively sought related literature, fostering the habit of active learning in daily practice; To ensure accurate diagnoses and appropriate treatment plans, residents sought consultation with the MDT, thereby fostering teamwork [8, 9]; To accelerate the publication of case reports, residents proactively facilitated the process from data collection to article submission, thus fostering their initiative in scientific research. Concurrently, residents often faced challenges during the case report completion, which inspired them to engage in continuous introspection. In the process, residents further digested this experience into their own inner knowledge and consciousness. For instance, if the submission process was impeded due to incomplete case data, they reflected on the reasons for their clinical history’s inadequacy. This prompted a commitment to thorough examinations and meticulous record-keeping in the future. When reflecting on the case uniqueness by comparing cases, residents developed their observation and pattern recognition skills [3]. These cultivated awareness, initiatives, habits, skills, and confidence are vital in residents’ clinical, scientific research and cooperative career endeavors, yielding significant long-term benefits [35].

This study, for the first time, verified the value of case reports in the professional development of residents in LMICs through qualitative research. The findings indicated that an educational approach grounded in case reporting could address the limitations of residency training including the lack of active learning in LMICs, utilize their abundance of cases and minimize external and internal pressures on residents in LMICs. Firstly, given the limited time and energy, the majority of residents in LMICs are unable to perform clinical duties and receive sufficient scientific research training at the same time [36]. In this context, case reports, being concise, can be published quickly without significant effort. Participants noted that this made case reporting training suitable for LMICs where residents face heavy clinical workloads and high stress. Secondly, unlike original studies such as randomized controlled trials and experiments, case reports can be completed with minimal research resources or financial expenditure [3, 13], thereby addressing the training need in countries with uneven distribution of standardized training sites and limited research funds. Thirdly, case reports help integrate clinical practice with scientific research theory, encouraging the use of evidence-based medicine in daily clinics [6]. Furthermore, this integrative approach avoids an overemphasis on the cultivation of scientific research ability, which may lead to excessive stress on residents and the production of “flashy” study results [37]. Finally, given the substantial population and plentiful case resources in most LMICs, such as China, enhancing resident competencies through case reporting training is feasible. Consequently, for LMICs that possess abundant case resources but are deficient in scientific research resources, this study proposes that case reporting be cultivated as a regionally distinct training and instructional platform for resident doctors. Future efforts should focus on creating specific training programs to tackle the challenges stemming from the lack of formal guidance in case report publication and create a safe and supportive learning environment for case report training [12, 30, 38].

### Strengths and limitations of the study

This qualitative study employs thematic analysis to examine the value of writing case reports in residency training, showcasing several key strengths. It captures the nuanced experiences and perspectives of resident physicians, providing rich insights often missed by quantitative studies. By focusing on case report writing as an educational tool, the research enhances understanding of its impact on residents’ clinical competencies, research skills and collaborative and communicative competencies. The rigorous methodology, including semi-structured interviews and iterative coding, bolsters the credibility of the findings. By highlighting the challenges, benefits and feasibility of case report writing, this research contributes valuable knowledge that can inform training programs and ultimately improve educational practices in medical residency.

However, the present study might suffer from selection bias in sampling strategy. Firstly, the study exclusively focused on residents who successfully published case reports, which may inflate perceptions of implementation feasibility while underestimating challenges. Interviewee accounts reveal several potential barriers to publication: (1) a lack of challenging cases due to departmental limitations; (2) insufficient data collection by attending physicians; (3) inadequate research writing skills; and (4) chance. These barriers suggest that hospitals with limited resources or poor educational focus may find it difficult to provide publishable cases for residents. Future research should therefore include interviews with residents who have not successfully published case reports to better understand barriers and necessary support. Additionally, interviews with supervisors should be conducted to identify the necessary conditions needed to support residents in completing their case reports. Secondly, the study’s scope was limited by insufficient departmental coverage, excluding residents from auxiliary departments like radiology and pathology. Case report specialization varies significantly across disciplines (e.g., dermatology emphasizing images, surgery focusing on pathology, ophthalmology focusing on examination results); additionally, supportive departments may primarily report diagnostic findings, while clinical departments can also highlight treatment innovations. These differences in focus, as discussed in the “Implementation Challenges” subtheme, can impact training workload. Nevertheless, case report writing is valuable across specialties for developing residents’ skills, guiding research, reinforcing clinical knowledge, and fostering independence. Future research should utilize snowball sampling and include residents from diverse departments and with varying publication experiences to mitigate selection bias. Finally, while pre-existing relationships between researchers and participants could have introduced bias through power dynamics or preconceived notions, the absence of direct hierarchical or collaborative relationships suggests this influence was minimal. Indeed, these established relationships may have facilitated more open and candid communication, enriching the data gathered.

## Conclusions

The present study indicated that case report publication experience can significantly improve the clinical professional, scientific research, collaborative and communicative competency of residents. It is suggested that case reports be utilized as a specialized platform for residency training in LMICs, where scientific research resources are limited, but there is an abundance of case material and the capacity to manage challenging cases. To address common barriers to adult learning, creating a safe and supportive learning environment for case report training is suggested. Additionally, further exploration and standardization of specific training programs to support case reporting are warranted.

## Electronic supplementary material

Below is the link to the electronic supplementary material.

Supplementary Material 1: Appendix A. Semi-structured interview guide; Appendix B. The participant demographics (N=15)

## Data Availability

The datasets generated and/or analyzed during the current study (i.e. the transcribed interview data) are not publicly available due to concerns regarding privacy, but are available from the corresponding author on reasonable request.

## Abbreviations

LMIC: Low- and medium- income country
CV: Curriculum vitae
GME: Graduate medical education
ICR: Intercoder reliability
