# Supplementary material for: The value of publishing case reports for the professional development of Chinese residents: a qualitative study
Source: BMC Med Educ. 2025 Jul 1;25:933. doi: 10.1186/s12909-025-07459-2 (PMC12210673; doi:10.1186/s12909-025-07459-2)
Supplement: Supplementary file 1 — Supplementary Material 1: Appendix A. Semi-structured interview guide; Appendix B. The participant demographics (N=15) [file 12909_2025_7459_MOESM1_ESM.docx]

**Appendix A: Semi-structured interview guide**

**Part A: Introduction**

We are particularly interested in your experiences and perceptions of case report publication. Could you please walk me through the process of case reporting, from figuring out which case to write about to the final publication? How do you feel about this experience?

**Part B: General information**

Gender:

Age:

Majority/ Department:

Training Hospital:

How many case reports have you published?

Among the case reports you have published, how many were written in English and published in sci-indexed journals? And how many were written in Chinese, your native language?

**Part C: Experiences and perceptions**

1 Under what circumstances/motivations did you decide to start writing a case report?

2 What process did you follow from the beginning of planning to write the case report to the final successful publication?

2.1 How did you find the clinical cases suitable for publication?

2.2 How did you communicate with patients to collect medical histories and obtain informed consent?

2.3 How did you search for literature?

2.4 How did you write the manuscript?

2.5 How did you select journals for submission?

2.6 How many journals rejected your manuscript before the case report was finally accepted it?

If more than once, how did your mentality change in the iterative process of manuscript submission and rejection?

2.6 How did you communicate with reviewers and editors during the revision process?

2.7 How did you seek help from supervisors and senior colleagues in completing the case report?

3. How has the experience of publishing case reports affected your clinical and scientific research skills?

4. What are the differences between writing case reports and other types of research in improving your post competency?

5 How did you balance the pressures you faced in completing the case report?

**Part D: Ending words**

I have finished all my questions. What else would you like to add to the above questions?

Thank you for your participation! After all the interviews and data analysis are completed, I may invite you to review and discuss the results of our study. I hope we can keep in touch.

**Appendix B. The participant demographics (N=15)**

| Interviewee | Age | Sex | Training stage | Training Hospital | City | Majority | Experience of case report publication | | Interview duration |
| --- | --- | --- | --- | --- | --- | --- | --- | --- | --- |
|  |  |  |  |  |  |  | In Chinese | In English |  |
| 1 | 27 | F | second stage | Peking University First Hospital | Beijing | Dermatology and Venerology | 2 | 1 | 72 |
| 2 | 24 | F | first stage | Peking University First Hospital | Beijing | Dermatology and Venerology | 0 | 1 | 41 |
| 3 | 27 | F | first stage | Peking University First Hospital | Beijing | Dermatology and Venerology | 0 | 1 | 34 |
| 4 | 22 | F | first stage | Peking University First Hospital | Beijing | Dermatology and Venerology | 2 | 1 | 33 |
| 5 | 33 | F | second stage | Peking University First Hospital | Beijing | Dermatology and Venerology | 4 | 3 | 34 |
| 6 | 30 | M | first stage | Peking University First Hospital | Beijing | Dermatology and Venerology | 0 | 3 | 24 |
| 7 | 28 | F | second stage | Peking University First Hospital | Beijing | Dermatology and Venerology | 0 | 6 | 31 |
| 8 | 29 | F | second stage | China-Japan Friendship Hospital | Beijing | Internal medicine (Nephrology) | 1 | 0 | 29 |
| 9 | 26 | F | first stage | China-Japan Friendship Hospital | Beijing | Chinese and Western Integrative Medicine (Nephrology) | 0 | 1 | 35 |
| 10 | 27 | F | first stage | China-Japan Friendship Hospital | Beijing | Chinese and Western Integrative Medicine (Pediatrics) | 1 | 0 | 37 |
| 11 | 26 | F | first stage | Peking University First Hospital | Beijing | Oncology | 0 | 1 | 37 |
| 12 | 28 | F | second stage | Xuan Wu Hospital | Beijing | Surgery | 0 | 2 | 20 |
| 13 | 25 | F | first stage | The First Affiliated Hospital of Zhengzhou University | Zhengzhou | Interventional Ultrasound | 1 | 0 | 28 |
| 14 | 28 | M | second stage | Peking University First Hospital | Beijing | Surgery | 1 | 2 | 19 |
| 15 | 28 | M | second stage | Beijing Hospital | Beijing | Internal Medicine (Pneumology) | 1 | 2 | 44 |
